# Motivation to Improve Mental Wellbeing via Community Physical Activity Initiatives and the Associated Impacts—A Cross-Sectional Survey of UK *parkrun* Participants

**DOI:** 10.3390/ijerph182413072

**Published:** 2021-12-11

**Authors:** Allison Dunne, Steve Haake, Helen Quirk, Alice Bullas

**Affiliations:** 1 Advanced Wellbeing Research Centre, Sheffield Hallam University, Sheffield S9 3TU, UK; s.j.haake@shu.ac.uk (S.H.); a.bullas@shu.ac.uk (A.B.); 2 School of Health and Related Research, University of Sheffield, Sheffield S1 4DA, UK; h.quirk@sheffield.ac.uk

**Keywords:** mental health, mental wellbeing, physical activity, *parkrun*

## Abstract

Participation in regular physical activity is a well-established strategy to support good mental wellbeing in adults with, and without, mental health conditions. The physical activity initiative *parkrun* is a free, weekly, timed, running and walking event which is attended by people from the local community of all abilities. The purpose of this study was to investigate the mental wellbeing of UK *parkrun* participants along with their motives for taking part and the impact of participation. Mental health conditions were self-reported in 2.5% of 60,000 respondents to an online survey of *parkrunners*, with the most prevalent being depression and anxiety. Those with mental health conditions were more motivated than those without to first participate in *parkrun* to manage their health conditions and improve their mental health. Those with mental health conditions were equally motivated to improve their physical health when compared to those without, and reported similar levels of improvement. Mental wellbeing scores for those with mental health conditions were close to the normal range, suggesting that engagement in *parkrun* may have had a role in limiting the effect of their illness. Community initiatives could replicate *parkrun’s* model, and use the potential for both mental and physical health improvement, as well as health condition management, as a motivation for participation.

## 1. Introduction

Good mental health and wellbeing are more than simply the absence of mental illness [1,2,3]: they have been described as a resource for everyday life [4]. The term mental wellbeing is often used interchangeably with positive mental health [5]. Subjective mental wellbeing relates to happiness, pleasure and life satisfaction along with personal growth and meaning in life [6]. Good mental wellbeing is a protective factor against developing future mental illness [7,8] and can even prevent or delay some physical health conditions [4,9].

Poor mental wellbeing can be a predictor of future chronic mental illness such as depression [10] and, to a lesser extent, anxiety disorders [11]. The World Health Organisation (WHO) report the prevalence of depression in the UK to be 4.5% and anxiety (including panic disorder and post-traumatic stress disorder (PTSD)) at 4.2% [12].

The determinants of mental wellbeing, which should be included in any health promotion strategy, are social, cultural, environmental and biological [4,9]. Physical activity has been shown to enhance mental health and wellbeing [13,14,15]. Being active in a natural environment [16,17,18] and in a group [19] can further contribute to good mental wellbeing. Social prescribing is often used to connect people at risk of poor mental wellbeing with community initiatives available in their local area [20,21] and an understanding of the motives of individuals to participate in physical activity may help with the selection and design of community initiatives [22].

### 1.1. parkrun

One community initiative which combines outdoor physical activity with social connectivity is *parkrun* (commonly written with a small ‘p’). *parkrun* is a registered charity which supports free, weekly, timed, physical activity events in the UK and 22 other countries worldwide [23]. The participants run or walk a 5 km route in an outdoor setting on Saturday mornings, with a 2 km option in some locations for children aged 4–14 years on Sunday mornings [24]. Volunteers from the local community organise the events, with support and equipment provided by the *parkrun* charity headquarters [25]. Many of the runners and walkers occasionally volunteer to support the events by undertaking roles such as timekeeping and marshalling [25]. Since the first *parkrun* in the UK in 2004, the event has now been organised in nearly 2000 locations internationally, with up to ten new *parkruns* starting every week [26]. This study took place in 2018, just after the launch of the social prescribing scheme called *parkrun* practice, which is coordinated in conjunction with the Royal College of General Practitioners [27]. Thus, almost all participants in this study attended *parkrun* through self-motivation rather than by a formal referral from a healthcare professional.

### 1.2. parkrun and Mental Wellbeing

No studies of *parkrun* have used an internationally validated scale to measure mental wellbeing although a range of scales related to mental health and wellbeing have been utilised. The subjective mental wellbeing of UK *parkrun* participants was explored in a cross-sectional study by Stevinson and Hickson [28] using a five-point scale designed by the researchers themselves. The same scale was used in a later study by Hindley [29]. Both studies showed that *parkrun* attendance frequency was positively associated with high mental wellbeing scores, although these were not compared with results for the general population [28,29]. Two studies showed that Perceived Stress Scale scores were reduced after *parkrun* participation both immediately after the event [30] and in the year following the initial registration with *parkrun* [31]. Grunseit, Richards and Merom [32] investigated one aspect of mental wellbeing which they described as personal wellbeing (life satisfaction) for *parkrunners* in Australia. Their results suggest that women’s personal wellbeing is connected to improved mental health, whereas men tended to experience wellbeing related to community connectedness. Stevinson and Hickson [31] used the Short Depression-Happiness Scale (SDHS) to measure the happiness and depression rating of *parkrun* participants, with improvements in mental health being maintained during the first year of *parkrun* participation.

### 1.3. Aim of This Study

There are no studies on *parkrunners* with mental health conditions compared to those without mental health conditions. In 2018, *parkrun* commissioned a survey to study the health and wellbeing of participants, including health conditions and mental wellbeing using the Short Warwick–Edinburgh Mental Wellbeing Scale (SWEMWBS) [33]. The aim of this study is to use the survey to compare the health and wellbeing of *parkrunners* with and without mental health conditions. The objectives are to determine the following:

1. What is their demographic?
2. What are their mental wellbeing scores?
3. What are their motives for first participating in *parkrun* as a runner or walker?
4. What are their perceived impacts of participation in *parkrun* as a runner or walker?

In answering these questions, this study seeks to provide guidance to those wishing to improve mental wellbeing using population-level physical activity initiatives.

## 2. Materials and Methods

Ethical approval for the *parkrun* Health and Wellbeing Survey 2018 was granted by Sheffield Hallam University Research Ethics Committee on 24 July 2018 (reference number: ER7034346). This study was supported by the *parkrun* Research Board. The reporting of this manuscript adheres to established standards for reporting internet-based surveys using The Checklist for Reporting Results of Internet E-Surveys (CHERRIES) [34].

### 2.1. Data Collection

#### 2.1.1. Inclusion and Exclusion Criteria

All UK *parkrun* registrants aged 16 and over (at the time of the survey) were invited to complete the online questionnaire. There was no paper version available. The survey was only available in the English language; there were no other explicit exclusion criteria.

#### 2.1.2. The Survey

This study used a cross-sectional online survey (the *parkrun* Health and Wellbeing Survey 2018). A power calculation was carried out prior to the survey which suggested that the sub-groups to be analysed would need to have >1000 participants if a moderate effect size was to be found with a significance of *p* < 0.05. A pilot study of 200 participants suggested a sample size of approximately 100,000 responses to get this fidelity. The survey was sent between 29 October 2018 and 3rd December 2018 to all adults in the UK aged 16 and over who were registered with *parkrun;* approximately two-thirds of these were active *parkrun* participants.

Data were collected using Qualtrics software (www.qualtrics.com, accessed on 29 October 2021). The full survey details and methods are described in earlier publications [35,36]. During the survey, respondents recorded their unique *parkrun* identification number which was used to match the survey responses with (1) demographic information (gender, date of birth and Index of Multiple Deprivation (IMD) from the postcode provided at registration) and (2) *parkrun* participation information (the date of registration with *parkrun* and the number of completed events). At the time of *parkrun* registration, the registrants were asked about their prior levels of physical activity. This was also matched to their survey responses.

The survey asked 47 questions relating to various aspects of mental and physical health and wellbeing. Responses to the following questions were used in this study:

- *parkrun* participation type: The *parkrun* model allows runners and walkers to volunteer at events if they wish to. It is also possible to volunteer without ever running or walking at the event. The participants were asked to tick the option that best described their participation in *parkrun*. Options were (1) volunteer, (2) runner/walker who volunteers and (3) runner/walker (who does not volunteer). This study does not include the data from those who only volunteer (option 1).
- Health condition, illness or disability: participants were asked “Are your day-to-day activities limited because of a health condition or disability which has lasted, or is expected to last, at least 12 months? Include conditions related to old age, sensory deficits, mobility problems, developmental conditions, learning impairments and mental health.” The possible responses were; Yes, limited a lot, Yes, limited a little, No, Do not know or would rather not say. Participants who indicated that they did have a health condition, illness or disability were then asked to select all the applicable conditions from a list of 56 options. A further option for Other was also available with a free text response (not included here)
- Motives for running or walking at *parkrun*: participants were asked, “What motivated you to first participate at *parkrun* as a runner or walker?” [they were asked to select a maximum of three answers out of a possible 20 motives]. Additionally, the choice Other allowed a free-text response (not reported here). The answer choices were displayed in randomised order to help reduce response bias.
- SWEMWBS: Mental wellbeing was measured using seven SWEMWBS statements introduced with “Below are some statements about feelings and thoughts. Please tick the box that describes your experience of each over the last 2 weeks”. The participant could choose from; None of the time, Rarely, Some of the time, Often, All of the time. Participants who responded to all seven statements were given a SWEMWBS score [33]. The validity and reliability of the SWEMWBS tool has been tested in English-speaking clinical and non-clinical adult populations in the UK [33,37,38] and clinical populations in Singapore [39].
- Perceived impact of running or walking at *parkrun*: participants were asked, “Thinking about the impact of *parkrun* on your health and wellbeing, to what extent has running or walking at *parkrun* changed:” [list of 15 impacts: much worse/worse/no impact/better/much better]. Additionally, the choice Other allowed a free-text response (not used here). The answer choices were randomised to help reduce response bias. Survey respondents who earlier indicated they had a health condition, disability or illness were asked one additional question to indicate the impact of running and walking on managing their health condition, disability or illness (much worse/worse/no impact/better/much better).

For the purposes of this report mental health conditions are defined as including the following conditions: depression, anxiety, panic attacks, post-traumatic stress disorder (PTSD), bipolar, attention deficit hyperactivity disorder (ADHD), schizophrenia and dementia (including Alzheimer’s disease) but excluding developmental disorders, organic mental disorders and neurological disorders.

### 2.2. Statistics

#### 2.2.1. Preliminary Analysis

The dataset of 60,000 respondents contained 38,072 runners/walkers and 21,928 runners/walkers who also volunteered. Data validation and preparation for statistical analysis were completed using Excel for PC (V16, Microsoft Corporation) and the initial handling and validation of raw data from the survey are described in a previous publication [36].

Some survey respondents did not answer all the questions on the survey or during *parkrun* registration; data tables include the relevant sample size (*n*) to reflect this.

#### 2.2.2. Statistical Analysis

Statistical analysis was used to compare demographic data for the respondents who reported mental health conditions with those who did not. As the conditions for parametric statistical methods were not met (not all results showed a normal distribution and there is no meaningful zero for the SWEMWBS) the data analysis was conducted using non-parametric statistical tools and reporting methods. SWEMWBS results were compared using Mann–Whitney U tests with effect size calculated using *r* = *Z*/√*n*, where *Z* is the standardised test statistic and *n* the number of ranked respondents [40]. The *χ*^2^ statistic was used to evaluate the differences between the motives for, and impacts of, *parkrun* participation between those who did and did not report mental health conditions. Effect size was calculated using *φ_c_* = √(*χ*^2^/*n*(*k* − 1) where *χ*^2^ is the test statistic, *n* is the number of respondents and *k* is the number of rows or columns (whichever is the smaller). As commonly used, an effect size = 0.1 was considered small, 0.3 medium, and 0.5 large [41]. Cross tabulation and statistical analysis were carried out using IBM SPSS Statistics for PC (v26). Statistical significance was set at *p* < 0.001.

## 3. Results

### 3.1. Demographics of Participants

Table 1 shows that 1496 participants reported a mental health condition; this represented 2.5% of the sample. It can be seen that 51.7% of the full sample were female, 9.6% came from the most deprived neighbourhoods and 5.1% reported less than one period of physical activity of 30 min or more per week at the time of *parkrun* registration (i.e., they were classed as inactive).

Those with a mental health condition were on average 4 years younger than those without (45.0 vs. 49.0 years), had been registered for a shorter amount of time (2.2 vs. 2.6 years), had done fewer *parkruns* in total (median number of runs 7 vs. 10 ), and had done fewer *parkruns* per year (12.6 vs. 14.5). All effect sizes were small.

**Figure 1:**
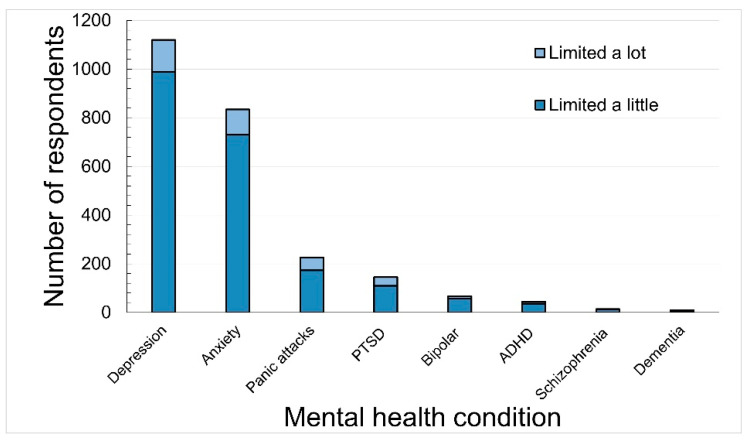
Number of respondents who selected specific mental health conditions (*n* = 1496). Note: dementia includes Alzheimer’s disease.

Figure 1 shows the mental health conditions reported by the respondents. Depression and anxiety were the most common mental health conditions reported by 1120 (1.9%) and 834 (1.4%) respondents, respectively. Some respondents reported more than one mental health condition with the maximum number of mental health conditions reported being six (Supplementary Material). A matrix showing the number of respondents with each condition is shown in Table 2. For those with depression, 584 (52%) also had anxiety, 184 (16%) had panic attacks and 89 (8%) had PTSD. Of the 834 with anxiety, 178 (21%) had panic attacks and 69 (8%) had PTSD.

### 3.2. Short Warwick–Edinburgh Mental Wellbeing Scores

Table 3 shows SWEMWBS scores for those with and without mental health conditions and who participated in *parkrun* as a runner/walker. The two largest conditions reported (by respondents who completed the SWEMWBS) were depression (*n* = 1040) and anxiety (*n* = 776) with scores of 19.3 compared to 25.0 for those without the conditions; these were significantly different but with small effect sizes of 0.16 and 0.14, respectively. Those with mental health conditions had a lower wellbeing score than those without for six of the conditions listed with the lowest score of 18.6 for those with panic attacks and PTSD compared to 25.0 for those without; there were small effect sizes of 0.06 and 0.08, respectively.

### 3.3. Motives for First Participating in parkrun for Those with a Mental Health Condition

The motives for first participating in *parkrun* as a runner or walker are shown in Table 4. The most reported motive for those with a mental health condition was *to improve my mental health*; the proportion choosing this motive was significantly larger for those with a mental health condition than for those without (49.0% vs. 12.0%; small effect size = 0.17). Similarly, those with a mental health condition were more likely than those without to choose *to improve my happiness* (10.8% vs. 6.6%; moderate effect size = 0.26) and *to improve or manage my health condition, disability or illness* (16.8% vs. 3.0%; small effect size = 0.12).

For the full sample, the top five motives were *to contribute to my fitness* (56.2%), *to improve my physical health* (37.0%), *to gain a sense of personal achievement* (26.9%), *to get a recorded time for a 5 k* (21.4%) and *to manage my weight* (19.8%). Those with a mental health condition were less likely than those without a health condition to choose *to contribute to my fitness* (36.4% vs. 56.7%) and *to get a recorded time for a 5 k* (12.6% vs. 21.6%), although effect sizes were small (less than 0.10); there was no significant difference for the three remaining top five motives.

### 3.4. Perceived Impact of Running or Walking at parkrun

Table 5 shows the perceived impact of running or walking at *parkrun* for those with and without a mental health condition with the proportions for *better* and *much better* combined. The remaining proportion for each measure was primarily *no impact* with the combined proportions for *much worse* and *worse* typically less than 1%. Seven of the measures showed statistical differences between those with and without a mental health condition (*p* < 0.001) with the largest difference seen for improvements to *mental health* (82.1% vs. 68.9%: small effect size = 0.05).

The largest proportion reporting improvement for the full sample was for *a sense of personal achievement*; a smaller proportion of those with a mental health condition reported improvement compared to those without (87.2% vs. 90.8%: small effect size = 0.02). The next most improved measures were *fitness* (89.3%), *physical health* (84.7%), *happiness* (78.8%) and *the amount of time spent outdoors* (74.1%); there were no significant differences between those with and without a mental health condition.

A larger proportion of those with a mental health condition reported improvements compared to those without for *confidence* (68.1% vs. 61.2%: small effect size = 0.02), *the ability to be active in a safe environment* (68.9% vs. 59.7%; small effect size = 0.03) and *overall lifestyle choices* (56.4% vs. 51.6%: small effect size = 0.02). A smaller proportion of those with a mental health condition reported improvements compared to those without to *the enjoyment of competing* (60.0% vs. 73.0%; small effect size = 0.04) and *how much you feel part of a community* (64.5% vs. 69.8%: small effect size = 0.02).

The final question in the series of impact questions was only asked to those who had indicated that they had a health condition. Respondents with mental health conditions were significantly more likely than those without mental health conditions to report improvements in their ability to manage the health condition since participating in *parkrun* (75.8% compared to 61.8%; small effect size = 0.13). The question did not specify the health condition so responses may have referred to non-mental health conditions (e.g., arthritis, asthma, and cancer).

### 3.5. Note on Results Section

The original data analysis segmented the results into age categories, gender, Index of Multiple Deprivation and activity level prior to *parkrun* participation. When the groups with and without mental health conditions were compared there were no significant findings within the segmented groups, so for clarity of reading we did not report each category here.

## 4. Discussion

In this self-selected sample of UK *parkrun* participants, 2.5% of respondents had a mental health condition: this was a lower prevalence of depression and anxiety than the published rates for the general population of the UK [12]. Those with a mental health condition were more likely to be female than male in a ratio 1.15:1 and more likely to be inactive at *parkrun* registration compared to those without mental health conditions. Those with a mental health condition tended to be younger, less likely to live in a deprived neighbourhood and did fewer *parkruns* per year than those without a condition. They were motivated to first participate in *parkrun* to manage their health conditions and improve their mental health and happiness; they perceived greater impact of *parkrun* for the first two of these motives.

Those with a mental health condition were less motivated to first participate in *parkrun* to improve their fitness although large proportions reported improvements to both this and their physical health that they attributed to *parkrun* participation. While those with a mental health condition were less motivated by competition, training for another event or getting a recorded time for a 5 k, 60% reported their enjoyment of competition to be improved since participating in *parkrun* (although this was still smaller than the 73% for those without a mental health condition). There were also large proportions reporting improvement to the measures relating to the supportive factors for good mental wellbeing such as spending time outdoors, being active in a safe environment and confidence. An understanding of the different motivations of people with and without mental health conditions to take part in physical activity will allow the developers of new community physical activity initiatives to support the engagement of both groups. Scales such as the Physical Activity and Leisure Motivation Scale (PALMS) [22] could be used in future research to develop a deeper understanding of the motivations of people with mental health conditions to participate in physical activity.

Mental wellbeing scores for the survey respondents showed some findings of note. The median SWEMWBS score for all survey respondents was 25.0. This is higher than the mean score for England (23.7 and 23.2 for males and females [42]) and the difference might be considered clinically significant [33]. At the time of the survey, those with a mental health condition reported SWEMWBS scores 5.8 to 6.4 points lower than those without a mental health condition. A low SWEMWBS score (20 and below) can be indicative of poor mental wellbeing; the survey respondents with mental health conditions had scores just below this threshold [43]. Given the perceived improvements by those with a mental health condition, particularly to measures related mental health, it is possible that without participation in *parkrun*, they may have experienced a deterioration of their mental wellbeing, putting them at greater risk of symptomatic mental illness.

Those with mental health conditions were equally motivated by physical health as those without and similarly large proportions (over 8 out of 10 respondents) report improvements to it that they attributed to *parkrun*. This is important since people with mental health conditions are at risk of physical comorbidities such as cardiovascular disease, type II diabetes mellitus and physical health conditions related to obesity [44,45].

### 4.1. Clinical Implications

People with symptomatic mental health conditions such as depression and anxiety often have low energy and motivation levels [12] and therefore may be less likely to attend organised physical activity events than those without mental health conditions [46]. It is also common for people with mental health conditions to experience social exclusion [47]. From previous research, it appears that *parkrun* is one possible activity to overcome these challenges and is a supportive activity for people with mental health difficulties [48]. The findings here further support this concept as the motivation to attend *parkrun* by people with mental health conditions as a way of managing their health conditions was high. The results suggest that *parkrun* is an acceptable option for health care practitioners to recommend via social prescription for mental health condition management. For the majority of the group with mental health conditions the initial motivation was not to improve physical health and fitness so this came as an added bonus shown through the impact results. People with mental health conditions often have poor physical health [4], so an intervention which can improve physical health while supporting mental wellbeing is an appropriate offering for social prescribers.

### 4.2. Comparison with Published Studies

This study used a validated mental wellbeing scale to support previous research which used other methods to describe the mental wellbeing of *parkrun* participants [28,29,32]. The body of research (including the results presented here) suggests that *parkrun* attendance can support good mental wellbeing in *parkrun* participants without mental health conditions. This study expands this concept to include those people with mental health conditions. Both groups reported different motivations to participate in *parkrun*; a finding which could be explored further when planning how to support people with differing mental health needs to engage with *parkrun.*

### 4.3. Implications for parkrun and Other Population-Level Events

The findings demonstrate that participation in *parkrun* could have a potentially important role in the management of mental health conditions. The evidence suggests that *parkrun* is perceived by those reporting mental health conditions to improve their mental wellbeing as well as physical health and fitness. Given the two-way relationship between physical and mental health this is an important finding [49]. These results would suggest that initiatives like *parkrun* could be used to help encourage people living with mental health conditions to increase their activity levels, spend time with others and increase the amount of time spent in the outdoors—all shown to contribute to good mental wellbeing. There is an opportunity here for initiatives such as *parkrun* to continue to support those with poor mental wellbeing and those living with mental health conditions to participate (e.g., through initiatives such as the *parkrun* PROVE project [50]). A social prescribing scheme such as the *parkrun* practice initiative [51] is another route for *parkrun* to engage with people with mental health conditions. Stevinson [52] suggested that the characteristics that made *parkrun* attractive to participants were that it was free, regular, can encourage socialisation and takes place in the outdoors. As well as these characteristics, the findings of this study suggest that the promotion or prescription of initiatives like *parkrun* should emphasise the potential benefits to mental health (as well as physical health), general wellbeing and the ability to manage health condition(s).

### 4.4. Limitations

While this study has the advantage of the use of a validated mental wellbeing scale in a large cohort, there are several potential limitations. The online data collection for the *parkrun* Health and Wellbeing Survey produced 60,000 fully completed surveys. This was lower than the planned sample size of 100,000, estimated following the pilot study. However, this was considered during segmentation of the sample to allow completion of a satisfactory statistical analysis. The cohort who completed the survey were older, more active, less likely to live in an area of deprivation and had completed more *parkrun* events per year than the whole *parkrun* population. The results of this analysis cannot necessarily be extrapolated to make claims about the complete *parkrun* population. The secondary data analysis uses a cross-sectional approach which does not allow for capture of baseline mental wellbeing scores. The question about motivation to participate in *parkrun* was asked retrospectively at the time of the survey; a more insightful method could be to ask the participants their motivation at the time of *parkrun* registration. The survey respondents self-reported their mental health conditions; these may not have been formally diagnosed in every case. The question about health conditions only collected information about conditions that limit daily activities and were long lasting. Information about previously diagnosed, but now well-controlled, mental health conditions was not collected. It should also be considered that maybe only those people in the local community with well-managed mental health conditions were able to participate in *parkrun* and/or take part in the survey.

## 5. Conclusions

Mental health conditions were prevalent in 2.5% of 60,000 respondents to a survey of *parkrunner*s, with the most prevalent being depression, anxiety, panic attacks and PTSD, often in combination. Those with mental health conditions tended to be younger and had participated in *parkrun* marginally less than those without. They were more motivated than those without mental health conditions to first participate in *parkrun* to improve their mental health and manage their health conditions; a larger proportion reported improvements to both. Those with mental health conditions were equally motivated to improve their physical health, weight management and fitness when compared to those without and showed similar levels of improvement. They were less motivated by competition, although 60% said their enjoyment of this was improved.

The mental wellbeing scores at the survey for those with mental health conditions were close to the normal range, suggesting that engagement in *parkrun* may have had a role in limiting the effect of their illness. This is an area which warrants further exploration. The characteristics of *parkrun* that make it a suitable social prescribing offer are that it is free, regular, can encourage socialisation and takes place in the outdoors. If other events want to support those with mental health conditions, they can replicate this model and both they and *parkrun* should emphasise both the mental and physical improvements that could be gained.

## Figures and Tables

**Table 1:** Demographics for the Health and Wellbeing Survey respondents at the time of the survey distribution (29th October to 3rd December 2018).

	Full Sample	With a Mental Health Condition	Without a Mental Health Condition	*χ* ^2^	*p*	*φ_c_*
*n*	60,000	1496	58,504			
Gender *n*	47,676	1170	46,506	90.9	<0.001	0.04
Female	51.7%	53.5%	51.7%			
Male	48.3%	46.5%	48.3%			
Index of Multiple Deprivation *n*	46,131	1127	45,004	0.48	0.924	0.003
Quartile 1	9.6%	9.1%	9.6%			
Quartile 2	20.4%	21.0%	20.3%			
Quartile 3	30.1%	29.9%	30.1%			
Quartile 4	40.0%	39.9%	40.0%			
Activity level at *parkrun* registration *n*	43,475	1069	42,406	3.65	0.456	0.009
<1	5.1%	5.6%	5.1%			
≈1	11.5%	13.1%	11.5%			
≈2	22.7%	22.2%	22.7%			
≈3	33.7%	32.4%	33.8%			
≥4	26.9%	26.8%	26.9%			
	Full sample	With mental health condition	Without mental health condition	*Z*	*p*	Effect size
Age *n*	43,621	1069	42,552			
Median	48.91	44.96	49.02	11.9	<0.001	0.06
Range: minimum to maximum	16.0–93.5	16.2–84.5	16.0–93.5			
Years registered *n*	60,000	1496	58,504			
Median	2.6	2.2	2.6	4.59	<0.001	0.02
Range: minimum to maximum	0–14.1	0–13.3	0–14.1			
Number of *parkruns* completed *n*	60,000	1496	58,504			
Median	10	7	10	5.65	<0.001	0.02
Range: minimum to maximum	0–612	0–612	0–582			
Number of *parkruns* per year *n*	34,609	841	33,768			
Median	14.4	12.6	14.5	4.86	<0.001	0.03
Range: minimum to maximum	0 to 55.6	0 to 50.3	0 to 55.6			

**Table 2:** Matrix showing the number of respondents who reported one or two mental health conditions. Note: dementia includes those reporting Alzheimer’s disease. The coloured cells show the number of people with a single health condition.

Limited Little and a Lot Combined	Depression	Anxiety	Panic Attacks	PTSD	Bipolar	ADHD	Schizophrenia	Dementia
Depression	1120	584	184	89	28	26	7	0
Anxiety		834	178	69	27	25	5	0
Panic attacks			226	2	8	5	2	0
PTSD				145	3	2	2	0
Bipolar					67	2	2	0
ADHD						44	1	0
Schizophrenia							14	0
Dementia								9

**Table 3:** Short Warwick–Edinburgh Mental Wellbeing Scale scores for those with and without specific mental health conditions who participated in *parkrun* as a runner/walker: statistical comparison used the Mann–Whitney U test.

	Full Sample		With Condition		Without Condition				
Condition	*n*	Median	*n*	Median	*n*	Median	*Z*	*p*	*r*
Depression	56,341	25.03	1040	19.25	55,301	25.03	39.0	<0.001	0.16
Anxiety	56,341	25.03	776	19.25	55,565	25.03	33.1	<0.001	0.14
Panic attacks	56,341	25.03	206	18.59	56,135	25.03	19.3	<0.001	0.08
PTSD	56,341	25.03	136	18.59	56,205	25.03	14.9	<0.001	0.06
Bipolar	56,341	25.03	60	18.92	56,281	25.03	9.23	<0.001	0.04
ADHD	56,341	25.03	39	19.25	56,302	25.03	6.88	<0.001	0.03
Schizophrenia	56,341	25.03	12	20.80	56,329	25.03	3.46	0.001	0.01
Dementia/Alzheimer’s disease	56,341	25.03	7	24.11	56,334	25.03	1.34	0.182	0.01
All	56,341	25.03	1389	19.25	54,952	25.03	43.1	<0.001	0.17

**Table 4:** Response to the question “What motivated you to first participate at *parkrun* as a runner or walker?” Participants (*n* = 59,243) were able to select up to three motives.

	Full Sample *n* = 59,282	With a Mental Health Condition *n* = 1496	Without a Mental Health Condition *n* = 57,786			
Motive	%	%	%	*χ* ^2^	*p*	*φ_c_*
To contribute to my fitness	56.2%	36.4%	56.7%	239.4	<0.001	0.06
To improve my physical health	37.0%	38.1%	37.0%	0.47	0.381	0.00
To gain a sense of personal achievement	26.9%	23.6%	26.9%	7.96	0.005	0.01
To get a recorded time for a 5 k	21.4%	12.6%	21.6%	69.1	<0.001	0.03
To manage my weight	19.8%	22.6%	19.7%	7.68	0.006	0.01
My friends, family or colleagues encouraged me to	15.2%	13.3%	15.2%	4.13	0.042	0.01
To train for another sport/event	14.2%	9.1%	14.3%	32.3	<0.001	0.02
To improve my mental health	13.0%	49.0%	12.0%	1742.2	<0.001	0.17
To feel part of a community	11.0%	9.8%	11.0%	2.41	0.121	0.01
To spend time outdoors	10.3%	8.3%	10.4%	6.49	0.011	0.01
To compete with others	9.8%	4.3%	9.9%	52.1	<0.001	0.03
To spend time with friends	7.7%	4.3%	7.8%	24.6	<0.001	0.02
To spend time with family	7.3%	3.8%	7.4%	27.2	<0.001	0.02
To improve happiness	6.7%	10.8%	6.6%	40.5	<0.001	0.26
It was part of a ‘couch to 5 K’ programme	5.3%	6.0%	5.3%	1.41	0.236	0.01
To meet new people	4.1%	4.9%	4.1%	2.46	0.117	0.01
To be active in a safe environment	4.1%	5.6%	4.0%	9.21	0.002	0.01
To improve or manage my health condition, disability or illness	3.4%	16.8%	3.0%	836.3	<0.001	0.12
To raise money for charity	0.5%	0.5%	0.5%	0.00	0.980	0.00
A health professional advised me to	0.3%	1.3%	0.3%	48.8	<0.001	0.03

**Table 5:** Response to the question “Thinking about the impact of *parkrun* on your health and wellbeing, to what extent has running or walking at *parkrun* changed your/the…” Proportions are for those reporting *better* and *much better*; comparison using *X*^2^ tests.

	Full Sample		With a Mental Health Condition		Without a Mental Health Condition				
Impact of Running or Walking at *parkrun*	*n*	Better and Much Better	*n*	Better and Much Better	*n*	Better and Much Better	*χ* ^2^	*p*	*φ_c_*
Sense of personal achievement	56,282	90.7%	1385	87.2%	54,890	90.8%	21.3	<0.001	0.02
Fitness	56,293	89.3%	1386	87.9%	54,868	89.4%	3.18	0.075	0.01
Physical health	56,241	84.7%	1388	83.4%	54,874	84.7%	1.68	0.195	0.01
Happiness	56,203	78.8%	1389	78.2%	54,825	78.8%	0.30	0.583	0.00
Amount of time spend outdoors	56,251	74.1%	1387	78.1%	54,864	74.0%	12.1	0.001	0.02
Enjoyment of competing	56,243	72.7%	1385	60.0%	54,874	73.0%	113.3	<0.001	0.05
How much you feel part of community	56,185	69.7%	1383	64.5%	54,827	69.8%	17.5	<0.001	0.02
Mental health	56,175	69.3%	1387	82.1%	54,848	68.9%	110.6	<0.001	0.04
Confidence	56,253	61.3%	1383	68.1%	54,806	61.2%	27.5	<0.001	0.02
Ability to be active in safe environment	56,224	59.9%	1388	68.9%	54,811	59.7%	47.4	<0.001	0.03
Number of new people you meet	56,247	57.5%	1386	57.0%	54,873	57.5%	0.18	0.673	0.00
Ability to control weight	56,218	52.3%	1382	50.0%	54,792	52.4%	3.13	0.077	0.01
Overall lifestyle choices (e.g., diet and smoking)	56,160	51.8%	1387	56.4%	54,862	51.6%	12.4	<0.001	0.02
Amount of time you spend with friends	6202	41.1%	1389	42.9%	54,752	41.1%	1.96	0.162	0.01
Amount of time you spend with family	56,235	27.7%	1385	24.4%	54,817	27.8%	7.91	0.005	0.01
* Ability to manage your health condition, disability or illness	5351	65.5%	1395	75.8%	3956	61.8%	88.65	<0.001	0.13

* response only for those with health conditions.

## Data Availability

The datasets supporting the conclusions of this article are stored in the Sheffield Hallam University Research Database (SHURDA) for access and in accordance with the Data Protection Act 2018 and the General Data Protection Regulation 2018. In the hope of ensuring the full research potential of the dataset, a copy of the anonymised data will be accessible to researchers for research purposes through the *parkrun* Research Board, as originally outlined in the participant information sheet.

## Supplementary Materials

The following are available online at https://www.mdpi.com/article/10.3390/ijerph182413072/s1. Table S1: Responses to the question “What is your health condition, disability or illness? Please select all that apply”.
