# Supplementary material for: Motivation to Improve Mental Wellbeing via Community Physical Activity Initiatives and the Associated Impacts—A Cross-Sectional Survey of UK parkrun Participants
Source: Int J Environ Res Public Health. 2021 Dec 11;18(24):13072. doi: 10.3390/ijerph182413072 (PMC8702167; doi:10.3390/ijerph182413072)
Supplement: Supplementary file 1 [file ijerph-18-13072-s001.zip › ijerph-1463718-supplementary.pdf]

Table S1. Responses to the question "What is your health condition, disability or illness? Please select all that apply"

Respondent ID	Gender	Age (years, rounded to nearest whole number)	Depression	Anxiety	Panic attacks	PTSD	Bipolar	ADHD	Schizo-phrenia	Dementia / Alzheimer's
78	F	52	Yes							
115	Unknown	56	Yes							
125	M	54	Yes	Yes						
148	M	63	Yes	Yes						
167	Unknown	51		Yes						
223	F	59	Yes	Yes						
273	M	55	Yes							
283	M	41	Yes							
370	M	65	Yes							
389	M	59	Yes	Yes			Yes			
395	M	49	Yes							
463	M	62	Yes				Yes			
550	F	55	Yes							
594	F	31	Yes	Yes						
643	F	41	Yes							
649	Unknown	34	Yes	Yes			Yes			
694	Unknown	27	Yes	Yes						
718	F	44	Yes	Yes						
764	Unknown	43	Yes	Yes						
785	F	64		Yes						
840	F	48		Yes						
879	M	51	Yes	Yes						
922	Unknown	35	Yes	Yes						
955	F	39	Yes							
1143	Unknown	62				Yes	Yes			
1186	F	42	Yes	Yes	Yes					

1248	F	48	Yes	Yes			
1356	M	28	Yes	Yes			
1423	M	58	Yes				
1513	Unknown	62				Yes	
1519	F	70	Yes	Yes			
1542	M	58	Yes	Yes			
1591	M	46	Yes				Yes
1607	M	58	Yes				
1658	M	51	Yes	Yes			
1675	F	47	Yes	Yes	Yes		
1734	F	40	Yes				
1743	F	51	Yes				
1963	M	54	Yes	Yes			
1999	Unknown	55	Yes				
2041	Unknown	47	Yes				
2060	F	51	Yes	Yes		Yes	
2067	F	35	Yes				
2135	M	53	Yes	Yes			
2216	M	55	Yes				
2246	M	34	Yes				
2253	F	59	Yes				
2260	M	44					Yes
2348	F	33	Yes	Yes			
2361	F	47	Yes	Yes			
2413	F	41	Yes				
2465	M	53					Yes
2568	F	37		Yes			
2581	M	28		Yes			
2751	Unknown	41	Yes				
2766	F	40	Yes	Yes			
2771	Unknown	31	Yes				
2806	Unknown	26		Yes			
2824	F	44		Yes			

2842	F	45	Yes						
2851	Unknown	34	Yes	Yes					
2854	Unknown	27	Yes	Yes					
2919	F	62	Yes						
2951	M	61	Yes	Yes					
2985	Unknown	17		Yes	Yes				
2987	Unknown	41	Yes		Yes				
3029	M	48	Yes						
3146	Unknown	32	Yes						
3164	F	50	Yes	Yes	Yes				
3181	M	32		Yes					
3188	Unknown	31		Yes					
3258	F	61		Yes					
3266	F	26	Yes	Yes	Yes				
3340	M	44	Yes						
3350	F	20		Yes					
3356	M	44							Yes
3371	Unknown	52				Yes			
3379	M	30							Yes
3439	Unknown	43		Yes					
3504	M	45	Yes						
3572	Unknown	54	Yes						
3585	F	27			Yes				
3666	Unknown	49	Yes	Yes					
3712	Unknown	missing	Yes					Yes	
3717	F	54	Yes	Yes		Yes			
3791	F	38	Yes	Yes		Yes		Yes	
3842	M	61		Yes					
3857	M	42	Yes						
3863	F	38	Yes	Yes	Yes	Yes			
3908	M	45	Yes	Yes		Yes			
3915	M	61	Yes						
3975	M	39	Yes	Yes					

4044	M	56	Yes							
4072	F	32	Yes	Yes						
4089	F	29		Yes						
4093	Unknown	43	Yes							
4110	F	48	Yes							
4114	Unknown	43		Yes						
4126	Unknown	30	Yes	Yes						
4139	F	45	Yes							
4294	F	51	Yes	Yes						
4314	M	51	Yes							
4345	M	50	Yes							
4370	M	60	Yes							
4378	M	26	Yes							
4424	F	37	Yes	Yes		Yes				
4429	M	58	Yes							
4478	F	43	Yes	Yes						
4527	M	47	Yes	Yes		Yes				
4546	F	41	Yes	Yes						
4580	F	42	Yes	Yes						
4595	M	55	Yes							
4730	M	59				Yes				
4772	Unknown	46	Yes							
4802	Unknown	24	Yes	Yes						
4896	M	59	Yes							
4913	Unknown	45	Yes							
5146	F	24				Yes				
5167	F	42	Yes	Yes	Yes					
5174	F	40		Yes						
5237	M	47	Yes							
5326	F	37	Yes							
5387	Unknown	60								Yes
5544	M	47	Yes							
5589	Unknown	48	Yes	Yes						

5622	M	44	Yes	Yes			
5695	M	18	Yes	Yes			
5720	F	59	Yes				
5921	M	54	Yes				
5928	M	28	Yes				
5938	M	40	Yes				
5976	F	48	Yes				
6110	F	30	Yes	Yes			
6331	Unknown	57	Yes				
6346	M	44					Yes
6402	M	39	Yes				
6475	M	42	Yes	Yes	Yes		
6476	F	38	Yes				
6483	M	52	Yes				
6506	M	39	Yes				
6523	Unknown	47	Yes	Yes	Yes		
6557	Unknown	26		Yes			
6597	M	46	Yes	Yes	Yes		
6698	F	26	Yes	Yes			
6759	F	18	Yes	Yes	Yes		
6899	F	31	Yes	Yes			
6907	F	25		Yes			
6910	F	55	Yes	Yes			
6923	F	61	Yes				
6928	F	36	Yes	Yes			
6953	M	36	Yes	Yes			
6985	F	58		Yes			
7007	F	46	Yes	Yes			
7034	M	36	Yes	Yes			
7041	F	46	Yes				
7080	F	42	Yes	Yes			
7104	M	40	Yes	Yes	Yes		
7113	F	25	Yes				

7153	F	47	Yes	Yes	Yes			
7165	F	57	Yes					
7166	M	58	Yes	Yes	Yes	Yes		
7187	M	53	Yes					
7224	F	63		Yes				
7286	F	34		Yes				
7303	F	42	Yes					
7341	F	45	Yes	Yes	Yes			
7445	F	38	Yes	Yes	Yes	Yes		
7516	F	66		Yes				
7565	F	52	Yes					
7585	F	39	Yes	Yes		Yes		
7607	F	43	Yes					
7614	Unknown	28	Yes			Yes		
7626	M	53	Yes					
7724	F	54					Yes	
7730	Unknown	21	Yes	Yes				
7744	M	48	Yes	Yes				
7763	Unknown	45				Yes		
7834	M	31	Yes	Yes				
7904	Unknown	52				Yes		
7918	F	30	Yes	Yes				Yes
7922	F	38	Yes	Yes	Yes			
8016	M	47	Yes	Yes				
8037	F	20	Yes	Yes	Yes			
8064	F	48	Yes					
8091	M	49	Yes					
8105	Unknown	38		Yes				
8177	M	53	Yes	Yes				
8217	F	50	Yes		Yes			
8296	F	31	Yes					
8362	F	40	Yes					
8530	Unknown	24	Yes	Yes				

8620	Unknown	46	Yes	Yes			
8621	M	54	Yes	Yes			
8641	M	65	Yes				
8642	F	54	Yes				
8663	Unknown	31	Yes	Yes			
8729	Unknown	64	Yes				
8778	F	29		Yes			
8811	M	27		Yes			
8839	M	50	Yes	Yes			
8861	F	37		Yes			
8976	M	47	Yes				
8979	F	55	Yes				
9004	F	26		Yes			
9030	F	55	Yes				
9070	F	35		Yes			
9080	F	59	Yes				
9104	F	55	Yes	Yes		Yes	
9107	F	59			Yes		
9189	M	38		Yes	Yes		
9282	Unknown	45		Yes			
9290	F	25	Yes	Yes			
9302	M	31	Yes	Yes			
9310	F	30	Yes	Yes			
9350	Unknown	38	Yes				
9421	M	49	Yes	Yes	Yes		
9449	F	51	Yes	Yes		Yes	
9482	F	46	Yes				
9521	F	48	Yes				
9557	M	43		Yes			
9574	F	29	Yes				
9589	F	55					Yes
9599	F	17	Yes				
9650	Unknown	19	Yes	Yes			

9654	F	52	Yes							
9655	Unknown	45	Yes	Yes						
9795	F	54				Yes				
9796	F	32		Yes						
9827	F	44					Yes			
9946	Unknown	60	Yes	Yes						
10013	F	22	Yes	Yes	Yes					
10149	M	65	Yes	Yes	Yes	Yes				
10179	M	51		Yes						
10190	F	36	Yes	Yes						
10318	F	52	Yes							
10442	F	45		Yes						
10456	M	62	Yes							
10501	Unknown	57	Yes		Yes					
10586	F	57	Yes							
10619	M	55	Yes							
10639	F	26		Yes						
10657	F	56	Yes	Yes	Yes					
10662	F	48	Yes	Yes						
10704	M	43	Yes	Yes	Yes				Yes	
10762	F	48		Yes						
10768	M	48	Yes							
10988	Unknown	57		Yes						
11045	M	43		Yes						
11120	Unknown	50	Yes	Yes						
11139	M	49	Yes	Yes			Yes			
11217	M	66		Yes						
11317	F	45	Yes							
11339	F	39	Yes	Yes	Yes					
11517	M	51	Yes							
11536	M	53	Yes	Yes						
11609	F	65			Yes					
11631	F	48	Yes							

11741	Unknown	19	Yes					
11805	M	46	Yes	Yes				
11848	Unknown	30		Yes				
11889	F	48	Yes					
11963	F	52	Yes					
12059	Unknown	43	Yes					
12091	F	55	Yes	Yes	Yes			
12098	M	37	Yes	Yes				
12099	M	61		Yes				
12103	F	62	Yes		Yes			
12151	F	46	Yes	Yes				
12208	M	35			Yes	Yes		
12213	F	52					Yes	
12252	M	57	Yes	Yes		Yes		
12299	M	53	Yes	Yes				
12325	F	33	Yes	Yes	Yes	Yes		
12379	Unknown	37	Yes	Yes				Yes
12418	M	57	Yes					Yes
12423	M	54		Yes				
12424	F	39	Yes					
12473	F	39		Yes				
12538	F	48				Yes		
12578	Unknown	48	Yes					
12584	M	40	Yes					
12601	F	33	Yes	Yes				
12627	M	40	Yes	Yes	Yes			
12641	F	47		Yes				
12646	F	34	Yes					
12678	F	46	Yes	Yes				
12849	F	52	Yes	Yes				
12850	Unknown	40					Yes	
12960	F	33	Yes					
13089	F	46		Yes				

13223	Unknown	56	Yes						
13248	F	48				Yes			
13343	M	44	Yes	Yes				Yes	
13349	F	44	Yes	Yes	Yes	Yes			
13508	F	18	Yes	Yes	Yes				
13528	F	56	Yes						
13539	M	40							Yes
13584	Unknown	24		Yes					
13657	F	49	Yes						
13761	F	67	Yes	Yes					
14030	Unknown	50	Yes						
14079	F	58		Yes	Yes				
14130	M	25		Yes					
14267	F	25	Yes	Yes	Yes				
14321	F	61	Yes	Yes					
14345	Unknown	53				Yes			
14424	F	27	Yes	Yes	Yes				
14456	F	46	Yes	Yes					
14471	M	33	Yes	Yes					
14472	F	34		Yes					
14511	F	32	Yes	Yes					
14569	Unknown	47	Yes	Yes					
14582	F	48	Yes						
14605	M	59		Yes					
14629	F	28	Yes	Yes					
14699	Unknown	26	Yes	Yes					
14709	F	29	Yes						
14860	F	41		Yes					
14955	F	38	Yes		Yes				
14970	M	44	Yes			Yes			Yes
14974	F	63	Yes						
14998	F	30	Yes	Yes					
15034	Unknown	53						Yes	

15066	F	33	Yes	Yes	Yes			
15136	M	58	Yes	Yes				
15275	F	28	Yes	Yes				
15292	F	40	Yes	Yes				
15302	F	50	Yes					
15331	F	49	Yes					
15420	F	40	Yes					
15477	Unknown	43					Yes	
15489	F	52	Yes	Yes				
15511	F	41	Yes	Yes		Yes		
15561	M	51	Yes	Yes				
15682	M	48		Yes				
15683	Unknown	23	Yes					
15742	F	57	Yes	Yes	Yes			
15808	F	56	Yes					
15835	F	45						Yes
15854	F	39	Yes	Yes				
15890	M	51	Yes					
15900	M	54				Yes		
15904	M	42	Yes					
15967	F	26	Yes					
15974	F	41		Yes		Yes		
15975	Unknown	25	Yes	Yes	Yes			
16050	F	47	Yes					
16082	F	46	Yes					
16149	Unknown	42	Yes					
16250	M	17						Yes
16276	Unknown	51					Yes	
16307	F	44	Yes	Yes				
16321	F	70			Yes			
16362	F	32	Yes					
16450	M	43		Yes				
16477	F	34	Yes	Yes				

16553	M	60							Yes
16558	F	44	Yes	Yes					
16615	F	22	Yes	Yes	Yes				
16642	M	44	Yes	Yes	Yes				
16718	M	46	Yes						
16795	F	36				Yes			
16798	M	64	Yes	Yes					
16806	F	26	Yes						
16877	F	20	Yes						
16951	F	21	Yes	Yes	Yes				
16969	F	47	Yes	Yes		Yes			
16996	F	49	Yes			Yes			
17044	M	56	Yes	Yes					
17052	F	29	Yes	Yes	Yes	Yes			
17076	F	17	Yes						
17080	F	48		Yes					
17124	F	45	Yes						
17171	F	28		Yes					
17182	F	34	Yes						
17194	F	48	Yes						
17238	M	40						Yes	
17419	F	65	Yes						
17436	M	54	Yes	Yes					
17464	Unknown	42	Yes						
17492	Unknown	25					Yes		
17526	F	29	Yes						
17563	M	27	Yes	Yes					
17748	M	42	Yes						
17888	Unknown	35	Yes						
17900	F	26	Yes	Yes	Yes				
18008	F	20	Yes	Yes					
18180	F	21		Yes		Yes			
18211	M	45	Yes	Yes					

18350	M	30				Yes		
18379	F	57		Yes	Yes			
18437	F	27	Yes	Yes				
18470	F	29	Yes	Yes				
18500	F	49	Yes	Yes				
18545	F	29	Yes	Yes				
18574	M	48	Yes				Yes	
18614	F	38	Yes			Yes		
18700	F	62	Yes					
18738	F	74	Yes	Yes				
18739	Unknown	52	Yes	Yes		Yes		
18817	F	missing					Yes	
18839	M	31	Yes					
18902	M	54	Yes	Yes			Yes	
18955	Unknown	27	Yes					Yes
18966	F	44						Yes
18997	M	35		Yes				
19080	F	45	Yes	Yes				
19114	M	59	Yes					
19175	F	21	Yes	Yes	Yes			
19194	M	43		Yes	Yes			
19208	F	30	Yes					
19311	F	39	Yes					
19411	M	36	Yes	Yes	Yes			
19467	M	22	Yes	Yes				
19468	F	22	Yes					
19488	Unknown	50	Yes	Yes				
19500	F	30	Yes	Yes				
19578	M	44		Yes				
19617	F	42	Yes	Yes	Yes			
19652	F	62	Yes	Yes	Yes			
19762	F	47	Yes					
19804	F	51	Yes	Yes				

19870	Unknown	17	Yes	Yes	Yes	Yes
19920	Unknown	26	Yes	Yes	Yes	
19924	F	35		Yes	Yes	
19935	M	31	Yes	Yes		
19946	F	32				Yes
19947	F	35	Yes	Yes		
19970	Unknown	55	Yes			
20010	M	53	Yes	Yes		
20011	Unknown	22	Yes	Yes		
20072	F	46	Yes	Yes	Yes	
20159	F	34		Yes		Yes
20210	M	51	Yes			Yes
20213	M	32	Yes	Yes		
20251	F	43	Yes	Yes	Yes	
20289	F	36	Yes	Yes	Yes	
20318	F	43	Yes		Yes	
20397	F	24		Yes		
20412	M	41	Yes	Yes	Yes	
20434	Unknown	28	Yes	Yes		
20441	F	37	Yes			
20496	F	66	Yes			
20545	Unknown	43	Yes	Yes	Yes	
20579	F	28	Yes	Yes		
20594	M	50	Yes			
20608	F	33	Yes			
20659	Unknown	20	Yes	Yes		
20714	M	26	Yes			
20734	F	32		Yes		
20813	F	25	Yes			
20839	F	19	Yes	Yes		Yes
20893	M	57	Yes	Yes		
21012	F	33		Yes		
21034	F	23	Yes	Yes	Yes	

21079	F	52			Yes	Yes		
21119	F	37	Yes	Yes				
21187	M	60	Yes					
21271	M	41	Yes					
21280	M	52	Yes					
21344	F	27		Yes				
21412	F	25	Yes	Yes				
21420	M	33	Yes					
21427	M	25	Yes	Yes				
21476	F	37	Yes	Yes				
21519	F	59	Yes		Yes			
21573	F	51		Yes				
21584	M	30	Yes	Yes				
21597	F	23	Yes	Yes				
21618	M	24		Yes	Yes			
21623	F	51	Yes	Yes				
21629	F	71		Yes				
21635	F	25		Yes			Yes	
21718	M	61		Yes				
21734	F	21		Yes				
21804	M	18	Yes	Yes				Yes
21811	F	49	Yes					
21891	F	70	Yes			Yes		
21924	Unknown	45		Yes				
21960	Unknown	59	Yes					
21995	M	55	Yes	Yes				
22007	M	54	Yes	Yes	Yes	Yes		
22013	M	61	Yes				Yes	
22087	F	38	Yes	Yes				
22166	F	25	Yes	Yes	Yes			
22188	Unknown	22	Yes	Yes	Yes			
22208	F	40	Yes			Yes		
22220	F	25		Yes				

22222	F	27	Yes					
22292	F	28	Yes	Yes				
22296	F	34	Yes	Yes				
22316	F	53	Yes					
22321	F	57	Yes	Yes				
22388	M	44				Yes		
22403	F	20	Yes	Yes	Yes			
22406	F	39		Yes				
22504	M	63	Yes	Yes				Yes
22511	F	23	Yes	Yes				
22545	Unknown	58	Yes	Yes				
22558	F	39	Yes	Yes				
22581	Unknown	43	Yes					
22730	M	36		Yes				Yes
22747	F	37	Yes					
22836	Unknown	29	Yes					
22851	M	54	Yes					
22863	F	42	Yes	Yes				
22869	F	43	Yes	Yes				
22889	M	33	Yes	Yes				
22910	F	47		Yes				
23010	Unknown	17	Yes					
23026	M	26		Yes		Yes		
23060	F	39	Yes					
23091	F	27		Yes				
23173	F	53	Yes					
23182	F	39	Yes		Yes			
23349	M	50	Yes					
23376	F	41		Yes				
23389	F	47	Yes					
23409	M	60		Yes				
23579	M	48	Yes					
23768	Unknown	34	Yes					

23840	M	73								Yes
23842	F	22	Yes	Yes						
23846	Unknown	33	Yes	Yes						
23866	Unknown	31	Yes							
23876	M	66	Yes							
23910	Unknown	46	Yes	Yes						
23922	F	44	Yes	Yes						
23983	M	41	Yes							
24020	M	34	Yes	Yes	Yes					
24040	F	46	Yes	Yes						
24050	F	32	Yes	Yes	Yes	Yes				
24058	F	54	Yes	Yes	Yes					
24158	F	59		Yes						
24320	F	48		Yes						
24390	F	44	Yes							
24394	F	46		Yes						
24430	M	63	Yes	Yes						
24496	F	30	Yes	Yes						
24509	F	51	Yes	Yes	Yes					
24554	M	60	Yes							
24639	M	31	Yes	Yes						
24716	F	44		Yes						
24752	M	64		Yes						
24761	M	55								Yes
24843	F	31		Yes						
24865	F	27	Yes	Yes		Yes				
25025	F	20			Yes					
25033	F	53	Yes							
25041	M	52	Yes	Yes						
25160	M	56				Yes				
25173	M	50				Yes				
25205	Unknown	53	Yes							
25326	F	41	Yes	Yes	Yes	Yes				

25418	Unknown	52	Yes	Yes				
25426	M	50	Yes			Yes		
25470	M	47	Yes	Yes				
25596	F	55					Yes	
25687	Unknown	40		Yes				Yes
25715	F	31	Yes					
25780	F	46	Yes			Yes		
25802	M	56	Yes					
25816	F	27	Yes					
25822	M	43				Yes		
25924	F	47	Yes	Yes	Yes			
26032	Unknown	54	Yes		Yes			
26069	F	50				Yes		
26088	F	17	Yes	Yes				
26146	F	48	Yes	Yes				
26192	F	39	Yes					
26202	M	31	Yes	Yes				
26204	Unknown	51				Yes		
26213	Unknown	27		Yes				
26244	M	56	Yes					
26255	F	44	Yes					
26281	F	42					Yes	
26339	Unknown	32		Yes				
26415	M	51	Yes					
26434	M	48	Yes	Yes	Yes			
26440	M	47				Yes		
26473	M	27	Yes					
26479	F	60		Yes				
26489	Unknown	64	Yes					
26490	F	50	Yes			Yes		
26537	F	23	Yes	Yes				
26543	M	29	Yes	Yes				
26583	M	43	Yes					

26601	F	55	Yes					
26753	F	62	Yes		Yes			
26800	F	50				Yes		
26826	F	36	Yes	Yes	Yes			
26842	Unknown	22	Yes	Yes				
26878	Unknown	44		Yes				
26911	M	59	Yes	Yes				
26934	Unknown	54	Yes	Yes				Yes
26972	Unknown	39	Yes					
26983	F	17	Yes	Yes	Yes		Yes	
27072	F	42		Yes				
27108	F	44	Yes	Yes	Yes	Yes		
27119	Unknown	30	Yes	Yes				
27183	Unknown	68	Yes					
27222	F	21	Yes	Yes	Yes			
27228	F	28	Yes					
27271	M	57		Yes				
27278	F	50	Yes					
27351	F	30	Yes	Yes				
27361	Unknown	55	Yes					
27478	F	22	Yes					
27512	M	37	Yes		Yes	Yes		
27517	F	57	Yes	Yes				
27529	F	58		Yes				
27573	F	55	Yes	Yes	Yes	Yes		
27614	F	31	Yes	Yes	Yes			
27639	F	50	Yes					
27732	F	47	Yes		Yes			
27766	M	38	Yes	Yes				
27783	F	38	Yes					
27804	F	56	Yes	Yes		Yes		
27839	F	36	Yes	Yes	Yes			
27856	F	43	Yes					

27879	M	42	Yes						
27906	M	45	Yes						
27918	F	35	Yes						
27969	M	32				Yes			
28022	F	56	Yes						
28083	M	24	Yes						
28112	F	36	Yes						
28222	F	29	Yes	Yes					
28253	F	48		Yes			Yes		
28271	F	46	Yes			Yes			
28302	F	47	Yes						
28361	Unknown	30	Yes	Yes					
28493	F	26	Yes	Yes					
28578	F	20	Yes	Yes	Yes				
28597	F	19	Yes	Yes	Yes			Yes	
28639	M	65	Yes			Yes			
28698	F	56						Yes	
28718	M	46	Yes						Yes
28722	Unknown	28	Yes						
28728	F	54	Yes						
28794	F	49	Yes						
28806	F	49						Yes	
28819	M	42	Yes	Yes		Yes			
28941	M	52	Yes						
28992	M	51	Yes	Yes					
29016	M	44	Yes	Yes					
29019	Unknown	45		Yes					
29047	F	24	Yes	Yes					
29083	F	42		Yes					
29172	F	44	Yes						
29226	Unknown	48	Yes	Yes		Yes			
29236	F	68	Yes						
29251	M	60		Yes					

29376	F	61		Yes						
29427	F	46	Yes	Yes						
29471	Unknown	51	Yes	Yes						
29526	Unknown	42		Yes						
29537	F	28	Yes	Yes						
29552	M	57	Yes							
29581	F	46	Yes							
29583	F	51	Yes	Yes						
29650	F	25	Yes	Yes	Yes					
29677	F	39		Yes						
29751	F	51	Yes	Yes		Yes				
29935	F	50	Yes							
29979	F	32							Yes	
30024	Unknown	48	Yes							
30038	M	43	Yes							
30172	M	62	Yes							
30182	F	24	Yes	Yes	Yes	Yes				
30273	F	58	Yes	Yes	Yes					
30347	Unknown	34	Yes			Yes				
30412	F	31	Yes							
30456	Unknown	59	Yes							
30598	F	48	Yes							
30626	F	28	Yes							
30703	F	58	Yes							
30718	M	55	Yes	Yes						
30735	F	49	Yes							
30835	Unknown	24	Yes							
30934	M	59								Yes
30958	M	57				Yes				
31044	M	37	Yes	Yes	Yes					
31087	F	51			Yes					
31100	M	26		Yes						
31120	M	68	Yes							

31205	F	45	Yes			
31284	M	39		Yes		
31349	F	44	Yes		Yes	
31378	M	35	Yes			
31496	F	31	Yes			
31500	F	46		Yes		
31551	M	47		Yes		
31585	F	24		Yes		
31733	F	54	Yes			Yes
31745	F	21	Yes	Yes		
31793	F	27	Yes	Yes		
32056	F	30	Yes	Yes		
32125	F	59		Yes		
32149	F	66	Yes			
32308	F	49	Yes	Yes	Yes	
32329	Unknown	44		Yes		
32524	F	32		Yes		
32625	Unknown	62	Yes	Yes	Yes	
32633	F	58	Yes			
32640	F	50	Yes	Yes		
32867	M	58	Yes	Yes		
32992	M	54	Yes	Yes		
33021	F	48	Yes	Yes	Yes	
33338	M	59	Yes	Yes		
33380	F	36		Yes		
33463	F	66	Yes			Yes
33496	Unknown	34	Yes	Yes		
33613	F	25	Yes	Yes	Yes	
33685	M	63		Yes		
34015	F	53	Yes	Yes		
34017	M	47	Yes			
34218	F	31	Yes	Yes		
34223	F	48	Yes	Yes	Yes	

34301	M	56	Yes	Yes						
34392	M	31	Yes	Yes						
34399	Unknown	54	Yes							
34410	F	44		Yes		Yes				
34458	F	46	Yes							
34595	Unknown	44	Yes							
34681	F	67	Yes							
34739	F	56	Yes							
34769	F	27	Yes	Yes						
34788	F	48		Yes	Yes					
34855	F	53		Yes						
34916	F	33	Yes	Yes	Yes					
35143	M	49				Yes				
35231	F	59	Yes							
35264	M	33	Yes	Yes						
35282	F	49	Yes				Yes	Yes		
35391	F	39	Yes	Yes	Yes	Yes				
35392	F	50	Yes							
35426	F	61	Yes							
35431	F	55	Yes							
35452	M	54		Yes						
35627	F	41	Yes							
35726	M	45	Yes	Yes						
35905	F	29	Yes	Yes	Yes	Yes				
35958	Unknown	40	Yes	Yes						
35990	M	47		Yes						
35991	Unknown	32	Yes	Yes						
36005	Unknown	43	Yes							
36212	F	37	Yes							
36405	M	56	Yes							
36490	F	20		Yes						
36506	F	54	Yes	Yes						
36534	Unknown	26	Yes	Yes						

36595	M	56		Yes			Yes
36645	M	34	Yes				
36785	F	37	Yes	Yes			
36789	M	67					Yes
36897	F	56		Yes			
36905	M	63				Yes	
36997	Unknown	48	Yes		Yes		
37037	Unknown	30	Yes	Yes			
37078	Unknown	31	Yes	Yes			
37084	F	21	Yes				
37124	Unknown	47	Yes	Yes			
37365	F	52	Yes	Yes	Yes	Yes	
37463	Unknown	33		Yes			
37473	M	21		Yes			
37562	Unknown	49					Yes
37688	Unknown	64					Yes
37819	F	43		Yes			
37854	M	53	Yes		Yes	Yes	
37886	F	28	Yes	Yes			
37948	F	21	Yes	Yes			
37950	Unknown	21	Yes				
38053	Unknown	18	Yes	Yes	Yes		
38103	F	32	Yes	Yes			
38385	Unknown	54	Yes				
38408	Unknown	45	Yes	Yes		Yes	
38502	F	65	Yes				
38505	M	53	Yes				
38521	M	65				Yes	
38723	M	57	Yes	Yes	Yes		
38821	M	51	Yes				
38934	F	40	Yes	Yes	Yes		
38960	M	70				Yes	
38976	F	26	Yes	Yes			

39056	Unknown	39	Yes		Yes			
39377	M	28	Yes	Yes				
39525	M	55	Yes					
39546	M	44		Yes				Yes
39572	Unknown	37	Yes					
39741	F	48	Yes	Yes				
39976	Unknown	26		Yes				
40237	Unknown	35		Yes			Yes	
40271	M	24	Yes	Yes				
40337	M	43	Yes					Yes
40358	F	30		Yes				
40409	F	46		Yes				
40416	Unknown	49	Yes					
40449	Unknown	52	Yes			Yes		
40465	F	24	Yes	Yes				
40476	Unknown	26	Yes	Yes	Yes			
40549	F	40	Yes					
40763	M	40	Yes					
40867	Unknown	62				Yes		
41016	F	41			Yes			
41138	Unknown	51		Yes				
41262	M	55	Yes	Yes	Yes		Yes	
41302	F	62	Yes					
41489	F	39	Yes	Yes	Yes			
41634	Unknown	64	Yes					
41654	Unknown	38	Yes	Yes				
41845	F	56	Yes					
41903	Unknown	22	Yes	Yes	Yes			
42214	Unknown	22	Yes	Yes	Yes			
42284	F	34		Yes				
42671	F	37	Yes	Yes				
42999	F	26		Yes				
43215	F	62		Yes				

43275	Unknown	45	Yes				
43340	F	54	Yes	Yes			
43345	M	36		Yes	Yes		
43746	F	32	Yes	Yes			
44059	M	44		Yes			
44226	Unknown	53	Yes	Yes			
44347	F	18	Yes	Yes	Yes		
44418	Unknown	53	Yes	Yes			
44543	F	37	Yes				
44662	Unknown	53	Yes	Yes			
44696	Unknown	22	Yes				Yes
44933	F	52	Yes				
44966	M	69	Yes	Yes		Yes	
45005	F	17		Yes			
45075	F	73	Yes				
45195	F	56	Yes	Yes		Yes	Yes
45279	F	44	Yes				
45370	Unknown	34	Yes	Yes	Yes	Yes	
45409	M	62				Yes	
45475	M	52	Yes	Yes			
45687	Unknown	29	Yes				
45692	M	53	Yes				
45809	Unknown	22	Yes	Yes			
45885	M	25	Yes				
45911	F	48	Yes				
45933	F	26	Yes				
45978	F	23	Yes	Yes	Yes		
46034	F	27	Yes				
46463	F	29		Yes			
46533	F	45		Yes			
46897	F	28	Yes	Yes			
47033	F	24	Yes	Yes			
47178	F	50		Yes			

47404	F	44		Yes					
47501	F	38	Yes	Yes	Yes				
47517	M	44	Yes						
47691	Unknown	26	Yes	Yes					
48001	M	49	Yes	Yes					
48614	Unknown	64		Yes					
48785	F	34	Yes	Yes		Yes			
48935	M	52	Yes						
49026	F	30		Yes					
49381	F	25	Yes	Yes					
49478	F	47	Yes						
49862	F	24		Yes					
49866	F	33			Yes	Yes			
49930	M	29	Yes	Yes				Yes	
50595	M	61				Yes			
50671	Unknown	24	Yes	Yes					Yes
50986	F	41	Yes						
51110	M	48							Yes
51270	F	50			Yes				
51882	Unknown	25		Yes					
51931	F	39	Yes	Yes	Yes				
52221	Unknown	33		Yes					
52344	M	69	Yes						
52620	F	45		Yes			Yes		
52653	M	50	Yes	Yes					
52748	Unknown	43	Yes	Yes					
53011	F	57		Yes					
53040	M	33	Yes						
53124	M	54	Yes	Yes					
53175	M	38	Yes	Yes	Yes				
53197	F	28	Yes			Yes			
53295	F	49				Yes			
53574	F	60				Yes			

53613	F	22	Yes					
53696	F	53			Yes			
53848	F	26	Yes	Yes	Yes			
53944	F	30	Yes		Yes			
54540	Unknown	45		Yes				
54603	Unknown	54	Yes	Yes	Yes	Yes		
55019	F	23	Yes	Yes				
55021	F	49	Yes					
55437	F	29	Yes	Yes	Yes			
55456	Unknown	45		Yes				
55466	Unknown	62	Yes	Yes	Yes			
56094	F	42	Yes					
56127	F	43	Yes	Yes			Yes	
56706	F	29	Yes	Yes	Yes			
56775	Unknown	53	Yes					
56904	Unknown	35	Yes	Yes		Yes		
57113	F	71	Yes					
57169	Unknown	45	Yes	Yes	Yes			Yes
57188	Unknown	52	Yes					
57469	F	31	Yes	Yes				
57570	Unknown	18	Yes					
58060	M	46	Yes					
58321	F	54	Yes					
58499	F	42	Yes	Yes				
58608	F	51	Yes	Yes				
58669	M	50	Yes					
59307	F	41	Yes					
59328	F	26	Yes					
59353	F	44	Yes					
59409	M	34	Yes					
59443	F	23	Yes	Yes				
59694	M	47		Yes				Yes
59969	M	56				Yes		

60078	F	61		Yes						
60137	F	49		Yes						
60144	M	36	Yes	Yes				Yes		
60200	M	69					Yes			
60826	F	25		Yes						
60847	F	56	Yes							
60850	Unknown	55	Yes							
60899	F	28	Yes	Yes		Yes				
60949	M	47	Yes	Yes						
60984	M	17	Yes	Yes						
60995	M	55	Yes	Yes						
61029	M	54		Yes						
61246	M	54	Yes							
61336	F	39	Yes							
61339	Unknown	37	Yes							
61373	F	73	Yes		Yes					
61385	Unknown	57	Yes	Yes	Yes					
61390	F	62	Yes	Yes						
61560	F	73	Yes							
61664	Unknown	53	Yes							
61869	F	52	Yes							
61934	Unknown	57	Yes	Yes		Yes				
62113	F	32		Yes	Yes					
62455	M	42		Yes						
62461	M	57				Yes				
62525	F	39	Yes							
63027	M	64								Yes
63300	F	46	Yes		Yes		Yes			
63721	F	30	Yes	Yes						
63819	Unknown	39	Yes							
63832	Unknown	56	Yes							
63884	M	61		Yes						
63897	F	65		Yes	Yes					

64164	M	60	Yes	Yes				
64321	F	55	Yes	Yes				
64377	M	43	Yes	Yes				Yes
64545	F	62	Yes					
64572	Unknown	53	Yes					
64854	F	59	Yes					
64960	M	68		Yes				
64963	F	58	Yes					
65038	M	28	Yes	Yes				
65715	F	35		Yes				
65958	M	29	Yes					
66168	F	49	Yes					
66447	F	52	Yes	Yes	Yes			
66553	F	35	Yes	Yes				
66646	M	57	Yes					
66748	M	48	Yes					
67023	F	42	Yes					
67143	Unknown	43	Yes					
67208	Unknown	53	Yes					
67216	F	23		Yes				
67337	Unknown	56	Yes					
67357	F	19	Yes	Yes				
67385	Unknown	57				Yes		
67474	F	48	Yes	Yes	Yes	Yes		
67601	M	37	Yes	Yes			Yes	
67703	Unknown	42	Yes	Yes				
67930	F	47	Yes	Yes				
67949	M	53	Yes		Yes			
68196	F	29	Yes	Yes	Yes			
68229	F	30	Yes	Yes	Yes			
68305	F	51					Yes	
68397	Unknown	51	Yes	Yes				
68468	F	55	Yes					

68494	M	46	Yes						
68530	M	60		Yes					
68531	Unknown	38		Yes					
68580	Unknown	40	Yes						
68652	F	60	Yes	Yes	Yes				
68754	F	40	Yes	Yes					
68761	Unknown	53		Yes	Yes				
68861	F	48		Yes					
68910	M	36	Yes	Yes					
69007	M	50	Yes			Yes			
69116	Unknown	24		Yes					
69193	M	55	Yes	Yes					
69205	F	16	Yes	Yes		Yes			
69280	F	44	Yes	Yes					
69327	M	46	Yes						
69523	F	32	Yes						
69564	M	51	Yes	Yes	Yes		Yes	Yes	Yes
69608	Unknown	36	Yes	Yes				Yes	
69625	M	30	Yes	Yes	Yes				
69656	M	31	Yes	Yes					
69671	F	35	Yes	Yes					
69750	F	28		Yes					
69782	F	39		Yes				Yes	
69786	Unknown	46	Yes	Yes			Yes		
69856	F	24	Yes						
69892	F	21					Yes		
69976	F	27	Yes	Yes	Yes	Yes			
70194	F	54		Yes					
70213	Unknown	56	Yes	Yes					
70329	M	64	Yes	Yes					
70809	F	22		Yes					
70865	Unknown	39	Yes	Yes					
71065	M	45	Yes						

71124	F	47	Yes	Yes						
71163	M	64	Yes			Yes				
71215	Unknown	49	Yes							
71220	F	48	Yes							
71247	Unknown	49	Yes							
71382	F	27	Yes	Yes						
71534	F	32	Yes	Yes		Yes				
71599	M	36	Yes		Yes					
71660	F	52	Yes	Yes						
71737	F	56	Yes							
72039	F	26	Yes							
72067	F	20	Yes	Yes						
72107	F	58	Yes	Yes						
72133	F	26		Yes						
72200	Unknown	30	Yes	Yes						
72249	M	71	Yes	Yes						
72339	M	36	Yes	Yes						
72384	F	59								Yes
72387	Unknown	53	Yes	Yes						
72429	F	53	Yes	Yes						
72464	M	66	Yes							
72635	M	56					Yes			
72643	M	23	Yes							
72734	F	51	Yes	Yes	Yes					
72960	F	42	Yes							
72997	F	52	Yes							
73124	Unknown	40	Yes	Yes	Yes					
73159	F	28	Yes	Yes	Yes					
73192	F	50	Yes	Yes	Yes					
73227	Unknown	48						Yes		
73333	F	44	Yes	Yes						
73620	F	50	Yes	Yes						
73693	M	40	Yes	Yes	Yes					

73786	F	47	Yes							
73841	M	27		Yes						
73873	F	25	Yes	Yes						
73880	Unknown	55	Yes							
73980	F	17	Yes	Yes						
74006	F	31	Yes							
74058	F	28	Yes	Yes						
74109	M	42	Yes							
74118	M	73	Yes							
74152	F	61	Yes	Yes						
74201	F	32	Yes	Yes						
74224	Unknown	56				Yes				
74235	F	40	Yes							
74263	F	52	Yes							
74331	F	36	Yes							
74344	F	33		Yes						
74459	F	51			Yes					
74620	F	42	Yes							
74677	Unknown	39	Yes	Yes						
74685	F	26		Yes						
74743	F	40	Yes	Yes						
74770	F	59	Yes	Yes						
74826	F	50	Yes	Yes		Yes				
74835	F	70								Yes
74871	F	25	Yes	Yes						
74874	F	18		Yes	Yes					
74893	F	39	Yes	Yes						
74944	Unknown	18	Yes	Yes	Yes					
74991	Unknown	27						Yes		
75074	F	65	Yes							
75129	M	61	Yes							
75211	F	61	Yes							
75284	F	53	Yes	Yes						

75322	F	52	Yes				
75339	F	39		Yes			
75426	F	45	Yes				
75452	M	54	Yes				
75598	F	21	Yes	Yes	Yes		
75604	F	31		Yes			
75821	F	58		Yes			
75830	M	35		Yes	Yes		
75861	M	64	Yes	Yes			
75903	M	50	Yes				
75909	F	51	Yes		Yes	Yes	
75919	F	57	Yes				
75999	Unknown	40	Yes	Yes	Yes		Yes
76088	M	46	Yes	Yes	Yes		
76097	Unknown	44	Yes				
76199	Unknown	29	Yes	Yes			
76209	M	52	Yes				
76262	M	49	Yes	Yes			
76300	F	33	Yes	Yes			
76342	F	37	Yes	Yes			
76385	F	45		Yes			
76535	M	45	Yes				
76536	M	59	Yes				
76569	M	46	Yes				
76656	Unknown	43	Yes	Yes			
76802	F	53	Yes				
76852	F	39	Yes	Yes		Yes	
77133	M	34	Yes				
77185	Unknown	63		Yes			
77224	F	52	Yes	Yes			
77432	Unknown	39	Yes	Yes			
77446	Unknown	27	Yes				
77530	Unknown	36	Yes				

77627	Unknown	18	Yes	Yes	Yes			
77830	Unknown	53	Yes	Yes	Yes			
77944	F	missing						Yes
77975	F	41	Yes					
78006	M	42	Yes	Yes				
78152	M	35	Yes					
78154	Unknown	61	Yes	Yes				
78257	F	48		Yes				
78425	M	34		Yes				
78486	F	61	Yes	Yes				
78728	F	44	Yes					
78785	F	16		Yes	Yes			
78915	F	54			Yes			
78984	M	25	Yes	Yes				
79019	F	45	Yes		Yes			
79042	Unknown	36	Yes	Yes	Yes			
79055	F	40	Yes			Yes		
79132	Unknown	52	Yes	Yes	Yes			Yes
79178	F	50	Yes					
79217	F	50	Yes					
79229	F	41	Yes					
79235	Unknown	41		Yes				
79291	M	61				Yes		
79294	F	55	Yes	Yes		Yes		
79347	F	16	Yes	Yes	Yes			
79358	F	44	Yes					
79367	Unknown	37	Yes	Yes				
79477	M	56	Yes	Yes				
79534	F	28		Yes				
79572	Unknown	38	Yes	Yes				Yes
79605	F	37		Yes				
79607	F	49	Yes					
79736	Unknown	38	Yes					

79831	Unknown	36		Yes			
79843	F	47	Yes				
79852	M	66	Yes				
79888	F	46	Yes	Yes			
79908	F	55	Yes				
79943	M	45	Yes				
79977	Unknown	51	Yes				
80076	F	74	Yes				
80107	M	61	Yes				Yes
80143	M	56	Yes				
80256	Unknown	29	Yes	Yes			
80290	Unknown	62	Yes				
80313	Unknown	52	Yes				
80359	Unknown	33				Yes	
80366	M	50		Yes			
80398	F	65	Yes				
80599	Unknown	40	Yes	Yes			
80675	M	52	Yes				
80679	M	48	Yes	Yes			
80686	F	58	Yes	Yes	Yes		
80723	F	56	Yes				
80726	M	38	Yes				
80840	F	36	Yes	Yes			
80933	Unknown	49		Yes			
80957	F	45		Yes			
81009	F	61		Yes	Yes		
81065	Unknown	39	Yes				
81084	M	57	Yes				
81122	Unknown	44	Yes				
81136	Unknown	52	Yes	Yes			
81255	F	47	Yes	Yes			
81348	F	63	Yes	Yes			
81377	F	53			Yes		

81405	Unknown	39	Yes			Yes			
81468	F	37		Yes					
81520	F	43	Yes	Yes					
81521	M	57						Yes	
81579	Unknown	61	Yes						
81606	Unknown	51	Yes						
81620	M	58	Yes						
81655	F	27	Yes	Yes	Yes				
81749	F	28	Yes						
81750	F	73	Yes						
81761	Unknown	47	Yes	Yes			Yes		
81798	F	51		Yes			Yes		
81813	F	32	Yes						
81821	F	35	Yes	Yes		Yes			
81863	F	37	Yes						
81885	F	53	Yes						
81910	M	56	Yes						
81920	Unknown	44	Yes						
81947	F	60	Yes						
81982	F	68	Yes						
82008	F	42		Yes					
82113	F	37	Yes	Yes					
82120	Unknown	43	Yes	Yes					
82148	M	31	Yes	Yes	Yes				
82155	Unknown	48	Yes						
82226	M	53	Yes		Yes				
82330	Unknown	44	Yes	Yes		Yes	Yes		Yes
82373	F	41	Yes	Yes					
82377	Unknown	53		Yes					
82483	M	47	Yes						
82631	M	38	Yes						
82639	Unknown	59	Yes						
82681	F	49		Yes					

82690	M	28	Yes	Yes				
82748	F	28		Yes				
82767	F	58	Yes					
82787	F	36	Yes				Yes	
82820	F	32	Yes	Yes				
82854	Unknown	40	Yes	Yes				
82857	Unknown	42	Yes					
82938	F	45	Yes					
82987	M	34						Yes
83019	M	48		Yes				
83023	F	54	Yes	Yes				
83078	Unknown	40	Yes					
83138	M	47	Yes	Yes				
83157	F	48		Yes				
83180	M	48			Yes			
83188	F	50	Yes	Yes				
83246	F	51	Yes					
83273	Unknown	57	Yes					
83304	Unknown	36	Yes					
83327	F	29	Yes					
83347	M	46	Yes	Yes				
83374	F	59	Yes					
83394	M	44	Yes					
83411	F	25	Yes					
83529	Unknown	54	Yes	Yes	Yes			
83577	F	46	Yes	Yes				
83586	M	53	Yes	Yes				
83591	M	49		Yes				
83614	M	60	Yes					
83675	M	57		Yes				
83721	M	50	Yes	Yes			Yes	
83781	F	26	Yes	Yes				
83793	M	36	Yes	Yes	Yes			

83886	M	54	Yes				
83910	F	40	Yes				
83957	F	49	Yes	Yes			
83999	M	55	Yes	Yes			
84001	Unknown	60	Yes				
84023	F	40	Yes				
84068	Unknown	48	Yes	Yes			
84095	Unknown	47		Yes			
84141	F	62				Yes	
84268	F	45	Yes		Yes		
84283	F	26	Yes	Yes			
84312	Unknown	49		Yes			
84327	F	36	Yes	Yes			
84420	Unknown	70			Yes		
84479	F	58	Yes	Yes			
84513	Unknown	40	Yes	Yes			
84582	F	39	Yes	Yes			
84663	Unknown	46	Yes			Yes	
84716	F	44	Yes				
84739	M	39	Yes				
84814	F	40	Yes				
84874	F	24					Yes
84880	Unknown	35		Yes			
84923	M	50	Yes	Yes			
84959	F	63	Yes				
85148	Unknown	24	Yes	Yes			
85174	Unknown	50				Yes	
85245	M	49	Yes	Yes			
85347	Unknown	42	Yes	Yes			
85403	F	32	Yes	Yes			
85519	Unknown	50	Yes				
85578	F	44			Yes		
85748	F	56	Yes	Yes		Yes	

85758	F	52		Yes					
85761	Unknown	43	Yes						
85818	F	52		Yes					
85898	F	54		Yes					
85917	F	45	Yes	Yes	Yes				
85921	Unknown	missing		Yes					
85998	F	58	Yes			Yes			
86007	Unknown	39	Yes	Yes					
86084	F	55	Yes						
86097	Unknown	54				Yes			
86126	Unknown	66		Yes					
86203	F	47	Yes						
86277	M	56	Yes						
86285	F	56	Yes	Yes	Yes				
86288	M	54	Yes	Yes					
86307	F	42	Yes						
86326	F	30	Yes	Yes					
86387	F	33	Yes						
86415	F	44			Yes				
86421	F	17		Yes					
86542	M	57	Yes	Yes					
86566	F	46	Yes	Yes	Yes				
86597	F	43	Yes						
86614	F	35	Yes						
86649	Unknown	38	Yes						
86673	F	40		Yes					
86696	F	56	Yes	Yes					Yes
86697	F	46	Yes	Yes	Yes				
86759	F	52	Yes	Yes					
86772	M	65	Yes						
86808	Unknown	50	Yes						
86863	F	37		Yes	Yes				
86864	F	36		Yes					

86881	Unknown	46	Yes	Yes			
86893	Unknown	31	Yes				
86923	Unknown	17		Yes			
86930	Unknown	45	Yes				
86946	F	49		Yes			
86951	Unknown	53	Yes	Yes			
86960	F	46	Yes	Yes			
87009	M	49					Yes
87207	F	41		Yes			
87228	F	50	Yes	Yes	Yes		
87246	F	46	Yes	Yes	Yes		
87309	Unknown	58	Yes				
87321	Unknown	40	Yes	Yes			
87458	F	41		Yes	Yes		
87460	F	27	Yes	Yes			
87483	M	38	Yes	Yes			
87498	Unknown	26	Yes	Yes	Yes		
87548	Unknown	38	Yes		Yes		
87598	F	27	Yes				
87634	F	30	Yes	Yes	Yes		
87654	F	49		Yes			
87686	Unknown	43		Yes		Yes	
87688	F	36		Yes			
87731	Unknown	39	Yes	Yes			
87742	Unknown	55	Yes				
87806	F	45		Yes			
87954	F	42	Yes	Yes			
88050	M	57			Yes		
88076	Unknown	43	Yes	Yes			
88086	Unknown	26			Yes		
88113	Unknown	40	Yes	Yes			
88118	M	73		Yes			
88157	F	31	Yes	Yes	Yes		

88179	F	45	Yes	Yes				
88215	M	70	Yes					
88228	F	48	Yes	Yes				
88231	Unknown	missing	Yes	Yes				
88268	Unknown	54	Yes	Yes				
88282	M	53					Yes	
88289	F	60	Yes					
88339	Unknown	38		Yes				
88348	Unknown	42	Yes					Yes
88437	Unknown	55				Yes		
88471	F	38	Yes	Yes				
88568	F	30	Yes	Yes				
88599	F	36	Yes	Yes				
88613	F	32					Yes	
88680	F	52	Yes					
88699	M	55	Yes					
88705	F	35	Yes	Yes				Yes
88736	Unknown	59					Yes	
88739	Unknown	25	Yes			Yes		
88749	Unknown	55	Yes	Yes				
88782	F	38	Yes	Yes	Yes			
88817	F	50		Yes				
88820	F	40	Yes	Yes				
88863	F	42	Yes	Yes		Yes		Yes
88898	F	40	Yes	Yes				
88912	F	33	Yes	Yes				
88927	M	55		Yes	Yes			
89143	F	72	Yes					
89180	F	46	Yes	Yes	Yes			
89246	Unknown	44		Yes				
89292	Unknown	42	Yes	Yes				
89336	F	59	Yes	Yes		Yes		
89349	F	64	Yes	Yes	Yes			

89374	F	45	Yes	Yes		Yes				
89376	Unknown	missing	Yes	Yes						
89429	Unknown	47	Yes							
89435	F	34	Yes	Yes	Yes					
89462	F	57	Yes	Yes						
89468	Unknown	48		Yes						
89479	M	67					Yes			
89489	M	27	Yes							
89557	F	28						Yes		
89627	F	56	Yes							
89669	F	52	Yes							
89711	F	36		Yes						
89713	F	55	Yes	Yes						
90076	Unknown	71	Yes		Yes					
90209	M	61	Yes	Yes				Yes		
90384	M	38	Yes							
90759	F	51	Yes							
90763	Unknown	50		Yes						
91199	F	44	Yes	Yes						
91484	F	43	Yes	Yes						
91547	M	39	Yes	Yes						
91600	F	58	Yes							
91741	Unknown	54	Yes	Yes						
91844	M	45	Yes							
91953	Unknown	38	Yes	Yes						
91956	M	82								Yes
91970	Unknown	56	Yes							
92031	M	46	Yes							
92151	F	32		Yes						
92231	F	28	Yes	Yes	Yes					
92234	M	46		Yes			Yes			
92242	F	43	Yes	Yes						
92444	M	55	Yes							

92529	Unknown	38	Yes						
92651	M	35	Yes	Yes					
92675	M	50	Yes						
92992	F	41	Yes						
93024	Unknown	24	Yes	Yes					
93068	M	43	Yes						
93230	Unknown	20		Yes					
93461	F	34	Yes	Yes	Yes			Yes	
93545	M	25	Yes	Yes					
93636	F	50	Yes						
93669	Unknown	26	Yes	Yes		Yes			
93720	Unknown	48	Yes	Yes					
93723	M	46							Yes
94066	M	36	Yes	Yes					
94516	Unknown	54	Yes						
94687	F	28	Yes	Yes	Yes		Yes		
94734	Unknown	28	Yes	Yes	Yes		Yes		
94800	F	51	Yes	Yes					
94847	Unknown	56	Yes	Yes					
95508	Unknown	34	Yes	Yes					
95591	F	44		Yes					
95733	F	46		Yes			Yes		
96134	Unknown	35	Yes						
96357	Unknown	41		Yes	Yes				
96361	F	43	Yes	Yes	Yes				
96500	F	49	Yes						
96541	F	49		Yes					
96590	F	47		Yes					
96611	M	41	Yes	Yes					
96631	M	54	Yes						
96968	F	47	Yes						
97019	Unknown	48	Yes	Yes	Yes		Yes		
97090	M	59				Yes			

97105	Unknown	60		Yes			
97149	F	29		Yes			
97591	F	49		Yes			
97770	M	29		Yes			
97870	M	53		Yes	Yes		
97879	F	47				Yes	
98005	Unknown	60	Yes				
98155	F	56	Yes				
98183	F	36	Yes	Yes			
98293	F	49	Yes	Yes	Yes		
98390	Unknown	55	Yes	Yes			
98402	F	53		Yes			
98682	M	17	Yes	Yes	Yes		
98810	Unknown	57					Yes
98957	F	55	Yes	Yes		Yes	
99110	M	42	Yes				
99271	Unknown	48	Yes				
99290	Unknown	62	Yes				
99447	Unknown	70	Yes				
99541	F	45	Yes	Yes			
99545	F	45	Yes				
99553	Unknown	55	Yes	Yes			Yes
99672	F	53	Yes				
99681	M	29	Yes	Yes	Yes	Yes	
99770	Unknown	54	Yes	Yes			
99776	M	66		Yes			
99796	F	40		Yes		Yes	
99934	M	46	Yes				
100003	M	55		Yes			
100037	F	53	Yes				
100135	Unknown	54	Yes	Yes	Yes		
100137	F	46	Yes	Yes			
100184	F	62	Yes				

100215	M	34	Yes							
100231	Unknown	44	Yes	Yes	Yes					
100251	F	39	Yes	Yes		Yes				
100257	Unknown	34	Yes							
100287	M	56		Yes						
100364	F	50		Yes						
100400	F	46					Yes			
100555	Unknown	49	Yes							
100612	F	54	Yes							
100650	Unknown	68					Yes			
100693	M	39	Yes							
100702	M	41	Yes	Yes	Yes					
100723	M	62	Yes							
100724	F	85								Yes
100756	F	44	Yes							
100779	Unknown	28	Yes	Yes	Yes					
100781	F	44	Yes							
100794	F	36	Yes	Yes						
